# A demographic profile of 7273 traumatic and non-traumatic spinal cord injured patients in Iran

**Published:** 2012-11

**Authors:** Vahid Eslami, Vafa Rahimi-Movaghar

**Affiliations:** ^a^Sina Trauma and Surgery Research Center, Department of Neurosurgery, Shariati Hospital, Tehran University of Medical Sciences, Tehran, Iran.; ^*b*^Research Centre for Neural Repair, Tehran University, Tehran, Iran.

## Abstract

**Background:**

To evaluate demographic proﬁle of traumatic and non-traumatic spinal cord injured (SCI) patients.

**Methods:**

Mobile rehabilitation teams gathered data in 20 out of 30 provinces in Iran. Of 8104 traumatic and non-traumatic SCI patients under coverage of the State Welfare Organization of Iran registered in the database, 7273 were included in the analysis.

The aggregate data on SCIs, including age, gender, place of residence, education level, marital status, etiology of injury, age at the time of injury, time passed since injury, level of injury, type of cord injury, having caregiver, and occupation were recorded.

**Results:**

Of 7273 patients, 5175 (71.1%) were male. At the time of the study, 46% were in the age group 20-40 years old, 34% were more than 40, and 20% were less than 20 years old. The residential place of 26% was in villages. 23.9% were illiterate, 6.9% had high school diploma or higher.

The distribution of cervical, thoracic, and lumbar levels of injury was 17.7, 24.4, and 57.9%, respectively. Overall, there were 49% married and 45.8% never married, while 1.4% patients were single because their partners had left them, 1.7% of partners had died, 1.9% had divorced, and 0.3% had remarried. At the time of the presentation of patients, 33% were 21-30 years-old, 17% were 31-40, and 16% were less than 20 years. About the type of cord injury, the paraplegia, paraparesia, quadriplegia, quadriparesia, and hemiparesia were present in 72.1, 12.5, 10.2, 4.0, and 1.1% of patients, respectively. Unemployment was reported in 55.6% of patients. However, 17% were unable to work, 7.1% had a job, and 3.4% were retired. Caregiver was not provided for 7.5% of them. The most prevalent causes of the injury were: trauma (57.4%), congenital (14.4%), tumors (4.4%), spinal degenerative disorder such as canal stenosis (2.2%), genetic (2.0%), infection (1.9%), scoliosis (1.1%), and miscellaneous (10.6%).

**Conclusions:**

These data will provide the information to guide future studies on SCI patients for better prevention and management of SCI patients.

**Keywords:**

Demography, Traumatic, Non-traumatic, Spinal cord injury, Etiology


					Volume 4, Suppl. 1 Nov 2012
Publisher: Kermanshah University of Medical Sciences
URL: http://www.jivresearch.org
Copyright:
Congress of Iranian Neurosurgeons, 14-16 November 2012, Kermanshah, Iran
Paper No. 47
A demographic profile of 7273 traumatic and non-traumatic
spinal cord injured patients in Iran
Vahid Eslami a
, Vafa Rahimi-Movaghar b,*
a
Sina Trauma and Surgery Research Center, Department of Neurosurgery, Shariati Hospital, Tehran University of Medical
Sciences, Tehran, Iran.
b
Research Centre for Neural Repair, Tehran University, Tehran, Iran.
Abstract:
Background: To evaluate demographic profile of traumatic and non-traumatic spinal cord injured (SCI) patients.
Methods: Mobile rehabilitation teams gathered data in 20 out of 30 provinces in Iran. Of 8104 traumatic and non-
traumatic SCI patients under coverage of the State Welfare Organization of Iran registered in the database, 7273
were included in the analysis.
The aggregate data on SCIs, including age, gender, place of residence, education level, marital status, etiology of
injury, age at the time of injury, time passed since injury, level of injury, type of cord injury, having caregiver, and
occupation were recorded.
Results: Of 7273 patients, 5175 (71.1%) were male. At the time of the study, 46% were in the age group 20-40
years old, 34% were more than 40, and 20% were less than 20 years old. The residential place of 26% was in
villages. 23.9% were illiterate, 6.9% had high school diploma or higher.
The distribution of cervical, thoracic, and lumbar levels of injury was 17.7, 24.4, and 57.9%, respectively. Overall,
there were 49% married and 45.8% never married, while 1.4% patients were single because their partners had left
them, 1.7% of partners had died, 1.9% had divorced, and 0.3% had remarried. At the time of the presentation of
patients, 33% were 21-30 years-old, 17% were 31-40, and 16% were less than 20 years. About the type of cord
injury, the paraplegia, paraparesia, quadriplegia, quadriparesia, and hemiparesia were present in 72.1, 12.5,
10.2, 4.0, and 1.1% of patients, respectively. Unemployment was reported in 55.6% of patients. However, 17%
were unable to work, 7.1% had a job, and 3.4% were retired. Caregiver was not provided for 7.5% of them. The
most prevalent causes of the injury were: trauma (57.4%), congenital (14.4%), tumors (4.4%), spinal degenerative
disorder such as canal stenosis (2.2%), genetic (2.0%), infection (1.9%), scoliosis (1.1%), and miscellaneous (10.6%).
Conclusions: These data will provide the information to guide future studies on SCI patients for better prevention
and management of SCI patients.
Keywords:
Demography, Traumatic, Non-traumatic, Spinal cord injury, Etiology
* Corresponding Author at:
Vafa Rahimi-Movaghar: Associate professor of Neurosurgery, Research Deputy, Sina Trauma and Surgery Research Center, Sina Hospital, Hassan-
Abad Square, Imam Khomeini Ave, Tehran University of Medical Sciences, Tehran, Iran. Phone: (+98) 915 342 2682, (+98) 216 6757010,
Fax: (+98) 216 675 7009, Email: v_rahimi@sina.tums.ac.ir , v_rahimi@yahoo.com, (Rahimi-Movaghar V.).

				

